# Activation of Platelet NLRP3 Inflammasome in Crohn’s Disease

**DOI:** 10.3389/fphar.2021.705325

**Published:** 2021-06-28

**Authors:** Ge Zhang, He Chen, Yifan Guo, Wei Zhang, Qiuyu Jiang, Si Zhang, Liping Han, She Chen, Ruyi Xue

**Affiliations:** ^1^ NHC Key Laboratory of Glycoconjugates Research, Department of Biochemistry and Molecular Biology, School of Basic Medical Sciences, Fudan University, Shanghai, China; ^2^ Department of Gastroenterology and Hepatology, Shanghai Institute of Liver Diseases, Zhongshan Hospital, Fudan University, Shanghai, China; ^3^ Department of Cardiology, Shanghai Institute of Cardiovascular Diseases, Zhongshan Hospital, Fudan University, Shanghai, China; ^4^ Shanghai Medical College, Fudan University, Shanghai, China

**Keywords:** NLRP3 inflammasome, Crohn’s disease, platelet hyperactivity, ROS, interleukin-1β

## Abstract

Patients with Crohn’s disease (CD) are inclined to have platelet hyperactivity and an increased risk of intestinal micro-thrombosis. However, the mechanisms underlying platelet hyperactivity in CD are not well understood. We investigated the assembly of platelet NLRP3 inflammasome in patients with active CD and its correlation with platelet hyperactivity. In this study, Real-time PCR and western blotting analyses uncovered that ASC, NLRP3, and active caspase-1 were significantly upregulated in platelets from patients with active CD compared with healthy subjects. As revealed by flow cytometry (FCM) and ELISA analyses, the levels of interleukin-1β in both serum and isolated platelets were elevated in patients with active CD. Co-immunoprecipitation and immunofluorescence experiments revealed an increased assembly of NLRP3 inflammasome in platelets from patients with active CD. In addition, higher levels of intracellular reactive oxygen species (ROS) were observed in these platelets by FCM. Furthermore, elevated levels of platelet P-selectin exposure and fibrinogen binding were demonstrated in patients with active CD by FCM. They were positively correlated with the protein levels of NLRP3 inflammasome components. Collectively, our results indicate that the ROS-NLRP3 inflammasome-interleukin-1β axis may contribute to platelet hyperactivity in active CD.

## Introduction

Inflammatory bowel disease (IBD) is a common chronic disease in the digestive system. It includes Crohn’s disease (CD) and ulcerative colitis (UC) (Roda et al., 2020). CD lesions are often located in the distal ileum and can also involve any location of the gastrointestinal tract, whereas UC lesions are usually confined to the colorectum (Zhang et al., 2014). In addition, CD lesions show transmucosal inflammation, while UC lesions are confined to the mucosal layer of intestinal cavity (Anderson et al., 2009). The annual incidence of CD ranged from 3–20 cases per 100,000 in the United States (Molodecky et al., 2012), and it has been increasing recently, especially in young adults aged 15–25 (Zhao et al., 2013). The etiology of CD has not been fully clarified yet. The current mainstream view believes that the occurrence of CD is related to genetics, environment, autoimmunity, nutrition and other factors (Singh et al., 2017; Turpin et al., 2018; Green et al., 2019; Khan et al., 2019; Schreiner et al., 2020). To date, there is no specific drug therapy for CD clinically, and the aim treatments, such as 5-aminosalicylic acid preparations, glucocorticoids and immunosuppressants are mainly used to control disease activity and delay disease progression (Chudy-Onwugaje et al., 2019; Na and Moon, 2019). However, the effects of drug treatment weaken with the progression of disease activity, and patients are prone to suffer from adverse clinical outcomes, such as allergy and drug resistance (Chudy-Onwugaje et al., 2019). Therefore, new therapies for CD are in urgent need, and understanding the pathogenesis of CD is of great basic and clinical significance.

Platelets contain many inflammatory factors stored in dense granules, α-granules or lysosomes, which are secreted when platelets are activated. These inflammatory factors have been reported to participate in a variety of physiological disorders, such as autoimmune diseases, atherosclerosis and tumors (Koupenova et al., 2018). Morowitz et al., 1968) first reported that platelet count and function in patients with active CD were abnormal, which were associated with the disease activity. Moreover, increased platelet count and platelet activity were unable to restore after the diseased intestinal segment was removed, indicating a crucial role of platelets in the progression of CD (Voudoukis et al., 2014). Hypercoagulability and thromboembolism due to platelet hyperactivity are common clinical complications in patients with active CD. Continuous platelet hyperactivity has also been observed to be associated with a prolonged disease course, and may be a pathogenic factor for disease recurrence. However, the mechanisms underlying platelet hyperactivity in CD are not well understood.

CD is characterized by aberrant immune responses and chronic inflammation in the gastrointestinal tract. Our previous studies have indicated a key role of intracellular NOD-like receptor (NLR) in autoimmune disease, and NLRP3, a member of NLR family, was activated in macrophages and colonic tissues of patients with active CD (Zhang et al., 2015; Zhen and Zhang, 2019). The activation of NLRP3 was closely correlated to the severity of the disease, which suggested that NLRP3 inflammasome might be a potential therapeutic target for CD (Tourkochristou et al., 2019). The NLRP3 inflammasome complex consists of the sensor component NLRP3, the adaptor component ASC, and the effector component caspase-1. As an effector protein, caspase-1 can cause maturation of interleukin-1β, an inflammatory factor, and further promote the occurrence of inflammatory damage (Kelley et al., 2019).

In addition to its presence in macrophages, the NLRP3 inflammasome has recently been identified in platelets (Hottz et al., 2013), and demonstrated to positively regulate hemostasis and arterial thrombosis. The deficiency of NLRP3 impaired platelet aggregation and spreading *in vitro*, and weakened hemostasis and thrombosis *in vivo*. The assembly of NLRP3 inflammasome also enhanced thrombosis in hypoxic environments (Gupta et al., 2017). Though it serves as a novel bridge between inflammation and thrombosis, the activity and role of NLRP3 inflammasome in platelets of CD remain unclear.

We investigated the activity of the NLRP3 inflammasome in platelets and its correlation with platelet hyperactivity in patients with CD.

## Materials and Methods

### Study Design

In this study, 40 patients with active CD (30 males and 10 females, aged 37 years (range, 17–67) were enrolled in Zhongshan Hospital, Fudan University from February 2019 to December 2020. Crohn’s disease was diagnosed according to the Lennard-Jones criteria based on clinical manifestations, endoscopic examination results and histological documentation. Exclusion criteria for enrollment included: 1) colorectal cancer or small intestine cancer; 2) severe infectious, neoplastic and autoimmune diseases; and 3) received chemotherapy or radiotherapy. In parallel, we enrolled 40 age-matched healthy controls [17 females, 23 males, aged 33 years (range, 23–47)] who were free of a history of gastrointestinal diseases, especially IBD. Baseline characteristics and laboratory findings are shown in Table 1. Among them, 17 CD patients and 17 matched healthy controls were selected for mRNA analysis in platelets; 38 CD patients and 38 matched healthy controls were selected for protein detection in platelets; 26 CD patients and 26 matched healthy controls were selected for P-selectin exposure, fibrinogen binding, and ROS levels analysis in platelets. In addition, 12 patients with active CD and 12 matched healthy controls were selected for co-immunoprecipitation and immunofluorescence experiments. Platelets from three random individuals were combined to generate four samples in each group. Experiments involving human volunteers were conducted strictly complying with the Declaration of Helsinki and approved by the Ethics Committee of Fudan University. Written informed consent was obtained from all participants.

**TABLE 1:** Baseline characteristics and laboratory findings in healthy controls and patients with active Crohn’s disease (CD).

Parameters	Health	CD	*p*
Age (years)	33.48 ± 5.67	37.15 ± 14.08	0.1238
Male/female gender (n)	23/17	30/10	0.098
CRP (mg/L)	0.68 ± 0.72	22.04 ± 45.90	**0.0044**
D-dimer (g/L)	0.31 ± 0.20	0.53 ± 0.77	0.0824
Fibrinogen (mg/dl)	326.75 ± 41.72	359.13 ± 132.25	0.1475
APTT (s)	28.46 ± 1.88	34.70 ± 37.46	0.2959
PT (s)	11.12 ± 0.84	12.21 ± 3.49	0.0583
ESR (mm/H)	11.68 ± 4.62	19.66 ± 19.41	**0.0051**
PLT (×10^9^/L)	239.43 ± 63.73	280.82 ± 83.67	**0.0183**
RBC (×10^12^/L)	4.51 ± 0.37	4.44 ± 0.75	0.5249
WBC (×10^9^/L)	6.44 ± 1.77	6.38 ± 1.93	0.7615
EO (%)	1.91 ± 1.34	2.73 ± 2.38	0.0617
BA (%)	0.41 ± 0.25	0.51 ± 0.27	0.265
NE (%)	59.91 ± 9.95	64.23 ± 10.61	0.0579
LY (%)	29.55 ± 6.63	24.31 ± 8.41	**0.0038**
MO (%)	5.69 ± 1.75	8.12 ± 2.70	**0.0001**
BA (×10^9^/L)	0.03 ± 0.02	0.03 ± 0.01	0.4239
EO (×10^9^/L)	0.16 ± 0.12	0.17 ± 0.15	0.7516
MO (×10^9^/L)	0.39 ± 0.11	0.52 ± 0.24	**0.0072**
LY (×10^9^/L)	1.61 ± 0.55	1.47 ± 0.55	0.1858
NE (×10^9^/L)	3.80 ± 1.34	4.15 ± 1.68	0.3315
MPV (fL)	11.42 ± 1.61	10.46 ± 1.00	**0.0027**
PCT (%)	0.24 ± 0.07	0.28 ± 0.07	**0.012**
P-LCR (%)	31.18 ± 9.81	28.63 ± 8.16	0.2099
PDW (%)	13.31 ± 2.49	12.17 ± 2.22	**0.0396**

Data are presented as n (%), or as median (interquartile range), as mean ± SD. Bold indicates that *p*-value is smaller than 0.05.

Abbreviations: Health, the healthy group; CD, the patients with active CD; APTT, activated partial thromboplastin time; PDW, platelet distribution width; PLT, platelet count; RBC, red blood cell; PCT, platelet crit; WBC, white blood cell; PT, prothrombin time; BA, basophil; P-LCR, platelet-large cell ratio; ESR, erythrocyte sedimentation rate; EO, eosinophil; LY, lymphocyte; NE, neutrophil; MO, monocyte; CRP, C-reactive protein; MPV, mean platelet volume.

### Blood Sample Preparation

Blood samples were obtained from patients with active CD or healthy volunteers once they were admitted to the hospital or on the day of physical examination. Washed platelets were separated as previously described (Zhang et al., 2020). In brief, blood samples were centrifugated at 600 × g for 3 min to obtain platelet-rich plasma (PRP). PRP was put into centrifugation at 680 × g for another 3 min. The pellets were re-suspended in Tyrode’s buffer (Guo et al., 2021), and the supernatants were used for ELISA experiments.

### Western Blotting Analysis

Platelets were harvested using lysis buffer supplemented with PMSF (Pierce, Germany) and a protease inhibitor cocktail (Roche, Switzerland). After protein concentration determination using a BCA kit (Beyotime, China), samples were loaded and electrophoresed for 90 min and then electro-transferred to PVDF membranes (Merck, Germany). The PVDF membrane was then blocked for 2 h with 5% BSA in TBST solution, and incubated with the indicated antibodies: rabbit anti-caspase-1 (CST, #2225S, 1:2,000), mouse anti-ASC (Santa Cruz, #sc-271054, 1:600), rabbit anti-NLRP3 (CST, #15101S, 1:2,000) and rabbit anti-GAPDH (CST, #8884S, 1:5,000) overnight at 4°C. After incubation with the corresponding HRP-conjugated secondary antibody (Thermo Scientific, 1:6,000), the membrane was scanned by chemiluminescence.

### Co-Immunoprecipitation

Washed platelets were lyzed in IP buffer (Shen et al., 2013). The supernatants were obtained by centrifugation at 10,000 × rpm for 12 min and precleared by adding protein A/G PLUS-Agarose beads (Santa Cruz Biotechnology, #sc-2003) for 4 h. The lysates were then incubated with rabbit anti-NLRP3 (CST, #15101S) or control IgG for 3 h, followed by incubation with beads overnight. The beads were then washed and boiled in loading buffer for 8 min, and centrifuged to pellets for western blotting.

### Immunofluorescence Staining

Washed human platelets were adhered to coverslips coated with poly-L-lysine (Sigma Aldrich, United States) for 40 min, washed thrice, fixed and permeabilized in Fixation/Permeabilization Solution (BD, United States), and then blocked in PBS solution with 2% BSA. The platelets were then incubated with goat anti-NLRP3 antibody (Novus, NB100-41104, 1:100), mouse anti-ASC antibody (Santa Cruz, sc-271054, 1:100), and rabbit anti-CD41 antibody (Abcam, ab134131, 1:300) at 4°C overnight, followed by incubation with 1:200 dilutions of relative secondary antibody for 1 h. A confocal laser scanner microscope (Leica TCS SP8, Solms, Germany) was used to obtain images that were then processed using LAS AF Lite software (Leica, Germany).

### Platelet Fibrinogen Binding, P-selectin and Interleukin-1β Detection

The levels of platelet activation *via* fibrinogen binding on platelets and surface P-selectin expression were explored as previously described (Peng et al., 2020). Briefly, PRP was fixed in PFA (4%) and then incubated with 100 mg/ml FITC-conjugated fibrinogen, FITC-conjugated interleukin-1β antibody (BD, #340515), PE-conjugated P-selectin antibody (Thermo Scientific, #12-0626-82), or APC-conjugated CD41 antibody (Biolegend, #303710) for 20 min in the dark. Each sample was recorded for 20,000 platelet events using a FACSCalibur flow cytometer (BD, Germany). CD41-positive cells were recognized as platelets.

### Real-Time PCR

RNA from platelets was extracted using the TRIzol reagent according to the standard manufacturer’s recommendations. The SuperMix for qPCR (Vazyme, China) was used to obtain complementary DNA. RT-PCR was performed with qPCR Master Mix (Vazyme, China) and analyzed using the ViiA7 RT-PCR instrument (Applied Biosystems, United States). The reference gene GAPDH was used for the normalization. The primers are listed in Table 2.

**TABLE 2:** The sequences of primers for RT-PCR.

Gene	ID	Primer sequence (5′-3′ orientation)	Amplicon (bp)
NLRP3	114548	Forward GGA​CTG​AAG​CAC​CTG​TTG​TGC​A	153
		Reverse TCC​TGA​GTC​TCC​CAA​GGC​ATT​C	
ASC	29108	Forward AGC​TCA​CCG​CTA​ACG​TGC​TGC	129
		Reverse GCT​TGG​CTG​CCG​ACT​GAG​GAG	
Caspase-1	834	Forward GCT​GAG​GTT​GAC​ATC​ACA​GGC​A	145
		Reverse TGC​TGT​CAG​AGG​TCT​TGT​GCT​C	
GAPDH	2597	Forward GGA​GCG​AGA​TCC​CTC​CAA​AAT	197
		Reverse GGC​TGT​TGT​CAT​ACT​TCT​CAT​GG	

### Measurement of Reactive-Oxygen Species

The levels of intracellular ROS in platelets was determined as previously described (Zhiyong Qi, 2020). In brief, Krebs-Ringer solution containing 1 mM calcium and 5 μM fluorescent probe DCFH-DA was used to resuspend washed platelets (1 × 10^8^ platelets/ml). The samples were analyzed using a FACSCalibur flow cytometer (BD, Germany).

### ELISA Detection of Interleukin-1β

An ELISA kit (Abcam, ab214025) was used to detect interleukin-1β levels according to the manufacturer’s recommendations.

### Statistical Analysis

Data are presented as mean ± SD. GraphPad Prism 6 (GraphPad Software, United States) was used for the data analysis. The one-sample Kolmogorov-Smirnov test was used to examine data normality. A two-tailed Student’s t-test was used to compare the difference between normally distributed continuous variables, whereas the Mann-Whitney *U*-test was used to compare abnormally distributed variables. Pearson’s chi-square test was used to compare differences in intergroup categorical data. Pearson and Spearman coefficients were used to determine the correlation between ROS levels or platelet NLRP3 inflammasome components and fibrinogen binding or P-selectin exposure on the platelet surface membrane. *p* < 0.05 was considered statistically significant.

## Results

### The Laboratory Findings of Healthy Controls and Patients With Active Crohn’s Disease

This study enrolled 40 patients with active CD and 40 matched healthy volunteers. Laboratory findings were summarized in Table 1. The active CD group comprised 30 males and 10 females and the mean age was 37.15 ± 14.08. The healthy group was composed of 17 females and 23 males and the mean age was 33.48 ± 5.67. The number of platelets was significantly higher (280.82 ± 83.67 × 10^9^/L) in patients with active CD than in healthy controls (239.43 ± 63.73 × 10^9^/L). While mean platelet volume (MPV, 10.46 ± 1.00 fL) and platelet distribution width (PDW, 12.17 ± 2.22%) of the active CD group were lower than the healthy group (MPV, 11.42 ± 1.61 fL; PDW, 13.31 ± 2.49%). In addition, there were higher levels of C-reactive protein (CRP, 22.04 ± 45.90 mg/L), erythrocyte sedimentation rate (ESR, 19.66 ± 19.41 mm/H) and plateletcrit (PCT, 0.28 ± 0.07%) in the active CD group compared with the healthy group (CRP, 0.68 ± 0.72 mg/L; ESR, 11.68 ± 4.62 mm/H; PCT, 0.24 ± 0.07%).

### Platelet NLRP3 Inflammasome Complex is Increased in Patients With Active Crohn’s Disease

**FIGURE 1:**
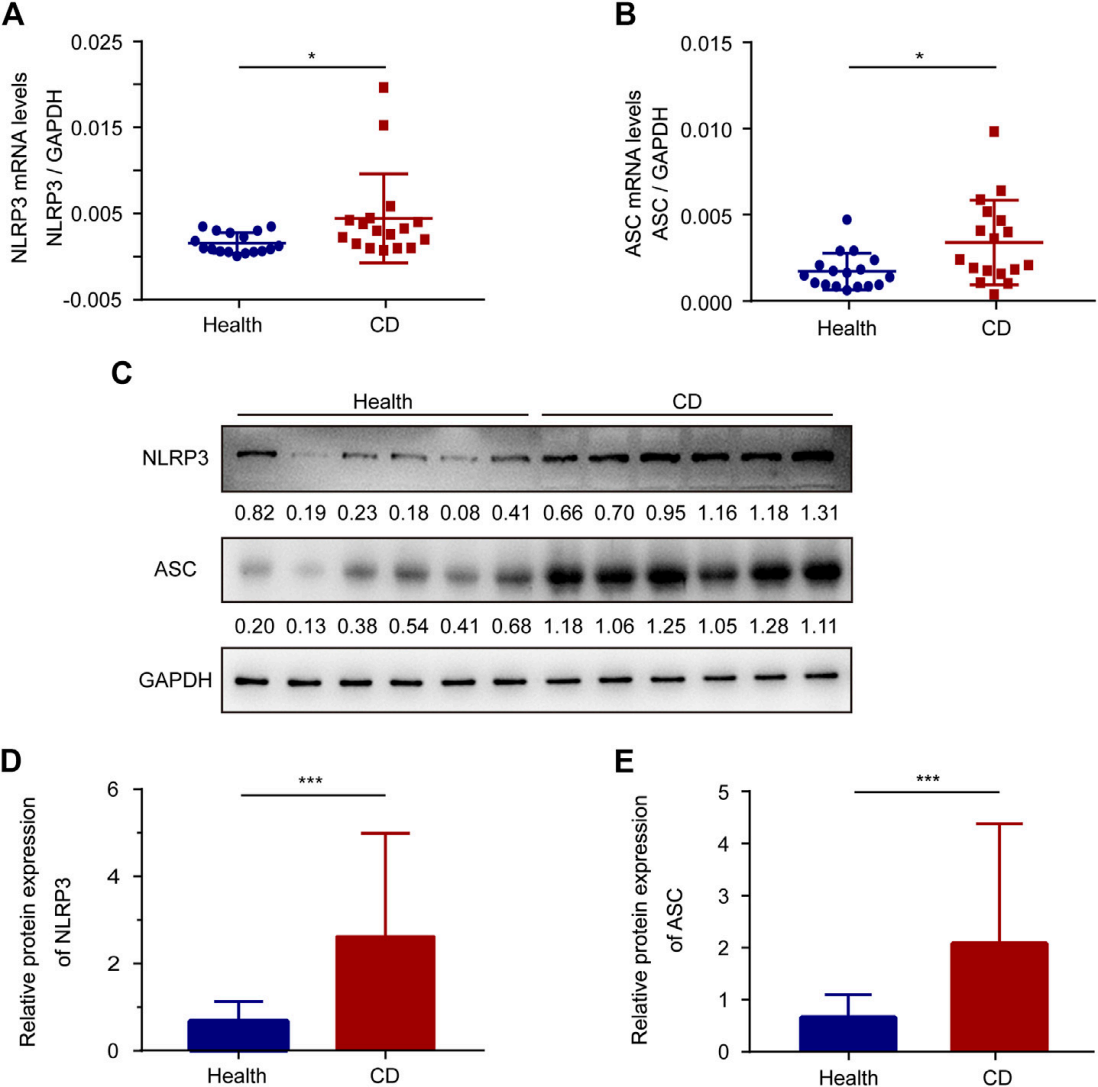
The expression of ASC and NLRP3 in platelets from the healthy group and patients with active Crohn’s disease (CD). **(A,B)** The mRNA levels of NLRP3 **(A)** and ASC **(B)** were determined by RT-PCR in platelets from the healthy group (*n* = 17) and patients with active CD (*n* = 17). **(C)** Western blotting was used to determine the protein levels of NLRP3 and ASC in platelets from patients with active CD and the healthy group. **(D,E)** The relative protein quantification of NLRP3 **(D)** and ASC **(E)** in platelets from the healthy group (*n* = 38) and patients with active CD (*n* = 38). Health, the healthy group; CD, the patients with active CD. Data are presented as mean ± SD, *p < 0.05, ***p < 0.001.

We explored the mRNA levels of sensor-NLRP3 and adaptor-ASC in platelets from the healthy group and the active CD group using RT-PCR (Figures 1A,B). The levels of sensor-NLRP3 and adaptor-ASC were increased 2.83-fold and 1.98-fold in platelets from patients with active CD compared with the healthy group, respectively. In addition, we also determined the protein levels of NLRP3 and ASC by western blotting (Figures 1C–E). A 3.85-fold increase in the NLRP3 protein levels and a 3.15-fold increase in the ASC protein levels were also observed in patients with active CD.

### The Assembly of NLRP3 Inflammasome is Increased in Platelets From Patients With Active Crohn’s Disease

**FIGURE 2:**
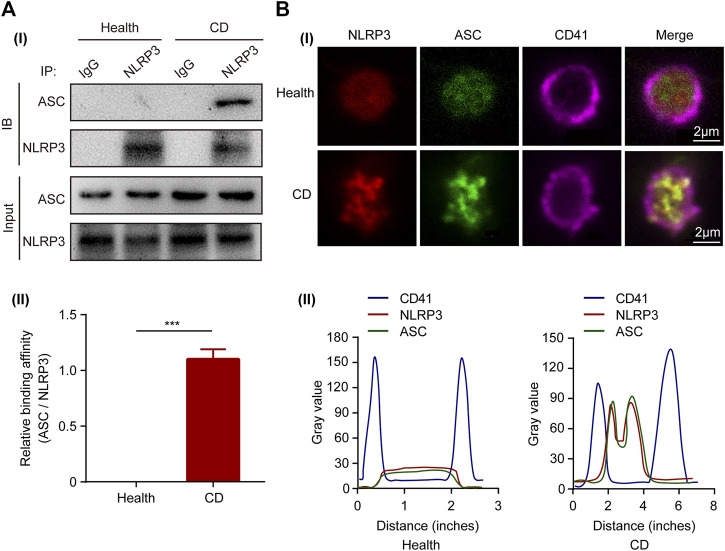
Activation of NLRP3 inflammasome in platelets from patients with active Crohn’s disease (CD). **(A)** Co-immunoprecipitation of ASC with NLRP3 in platelets from the healthy group and patients with active CD **(i)**. Quantification of the interaction between ASC and NLRP3 in platelets was below (*n* = 3), **(ii**). **(B)** Immunofluorescence staining showed colocalization of ASC (green) and NLRP3 (red) in platelets from patients with active CD **(i)**. The experiment was repeated three times. Intensity traces were plotted below **(ii)**. Scale bars were 20 μm. Health, the healthy group; CD, the patients with active Crohn’s disease. Data are presented as mean ± SD, ***p < 0.001.

Upon activation, the sensor-NLRP3 interacts with the adaptor-ASC via the PYD domain to form inflammasome complex. To investigate the activity of NLRP3, we determined the interaction between ASC and NLRP3 by co-immunoprecipitation in platelets. The binding of ASC to NLRP3 was observed in platelets from patients with active CD, but not in the healthy controls (Figure 2A). Confocal immunofluorescence staining further demonstrated that NLRP3 (red) and ASC (green) were diffusely distributed in the cytoplasm of platelets from healthy controls, while they aggregated into clusters and were co-localized together in platelets from patients with CD (Figure 2Bi). The colocalization of NLRP3 with the adaptor-ASC was analyzed using intensity traces (Figure 2Bii). Collectively, these results indicate an increased assembly of platelet NLRP3 inflammasome in patients with active CD.

### The Effector-Caspase-1 of NLRP3 Inflammasome Is Activated in Platelets From Patients With Active Crohn’s Disease

**FIGURE 3:**
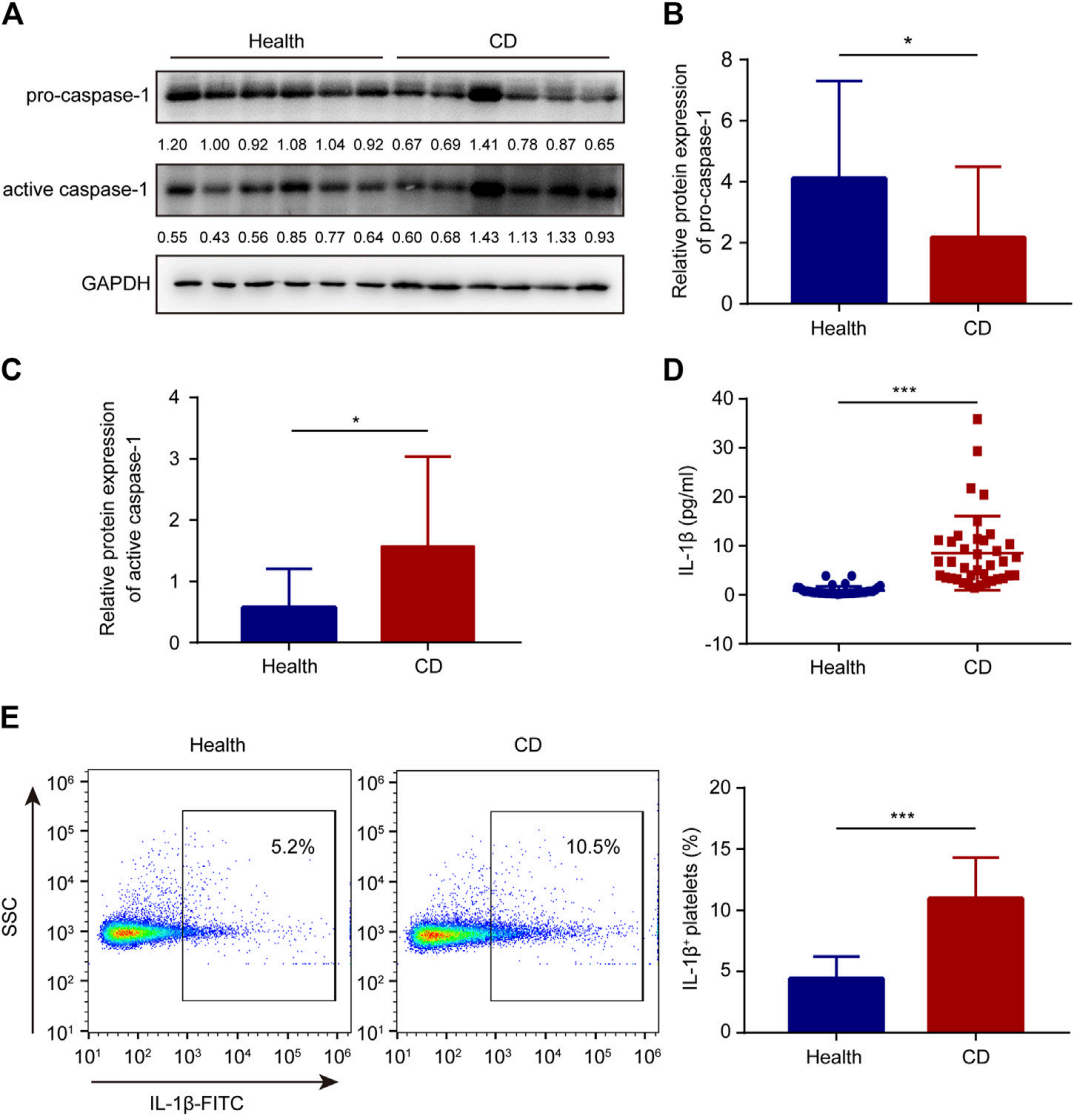
Increased interleukin-1β and active caspase-1 in platelets from patients with active Crohn’s disease (CD). **(A)** The protein levels of caspase-1 and pro-caspase-1 in platelets from the healthy group and patients with active CD. **(B,C)** The relative protein quantification of pro-caspase-1 **(B)** and active caspase-1 **(C)** was determined (*n* = 38). **(D)** Serum interleukin-1β levels were determined in the healthy group (*n* = 38) and patients with active CD (*n* = 38) by ELISA. **(E)** The ratio of interleukin-1β-positive platelets was determined in the healthy group (*n* = 38) and patients with active CD (*n* = 38). Health, the healthy group; CD, the patients with active CD. Data are presented as mean ± SD, *p < 0.05, ***p < 0.001.

The cleavage of pro-caspase-1 is triggered by activated NLRP3 inflammasome. When active caspase-1 is released, it cleaves pro-interleukin-1β and generates mature interleukin-1β, a bioactive form. Therefore, western blotting was used to determine the protein levels of active caspase-1 and pro-caspase-1. In platelets, the active caspase-1 protein levels were evidently higher in patients with active CD than in the healthy group (Figures 3A,C). Meanwhile, the pro-caspase-1 protein levels were lower in patients with active CD than in the healthy group (Figures 3A,B). In addition, there was no significant difference in caspase-1 mRNA levels between the healthy subjects and patients with active CD (Supplementary Figure S1). Active caspase-1 is conducive to the generation of interleukin-1β. Increased serum interleukin-1β levels were observed in patients with active CD, compared to the healthy group (Figure 3D). Interestingly, increased number of interleukin-1β-positive platelets was also observed in patients with active CD (10.98 ± 3.34%) compared with the healthy group (4.42 ± 1.81%) (*p* < 0.001) (Figure 3E).

### Reactive Oxygen Species is Increased in Platelets From Patients With Active Crohn’s Disease

**FIGURE 4:**
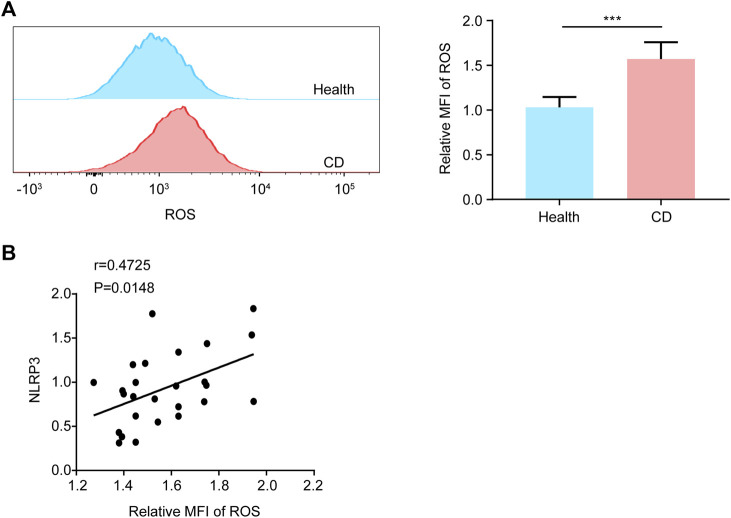
Increased ROS generation in platelets from patients with active Crohn’s disease (CD). **(A)** We explored the ROS generation by flow cytometry in platelets from the healthy group (*n* = 26) and patients with active CD (*n* = 26). The relative quantification was at right. **(B)** The NLRP3 protein levels were positively correlated with ROS levels in platelets from patients with active CD (*n* = 26). Health, the healthy group; CD, the patients with active CD. Data are presented as mean ± SD, ***p < 0.001.

Considering that ROS is of great importance in activation of NLRP3 inflammasome (Ratajczak et al., 2019), we evaluated the ROS levels in platelets by FCM. Compared with the healthy group, the intracellular ROS levels of platelets were increased 1.52-fold in patients with active CD (Figure 4A), indicating that elevated ROS generation might contribute to the formation of platelet NLRP3 inflammasome. Most importantly, we found that ROS levels were correlated with the NLRP3 protein levels in platelets from patients with active CD (Figure 4B).

### Reactive Oxygen Species Levels or NLRP3 Inflammasome Components Positively Correlate With Platelet Hyperactivity in Patients With Active Crohn’s Disease

**FIGURE 5:**
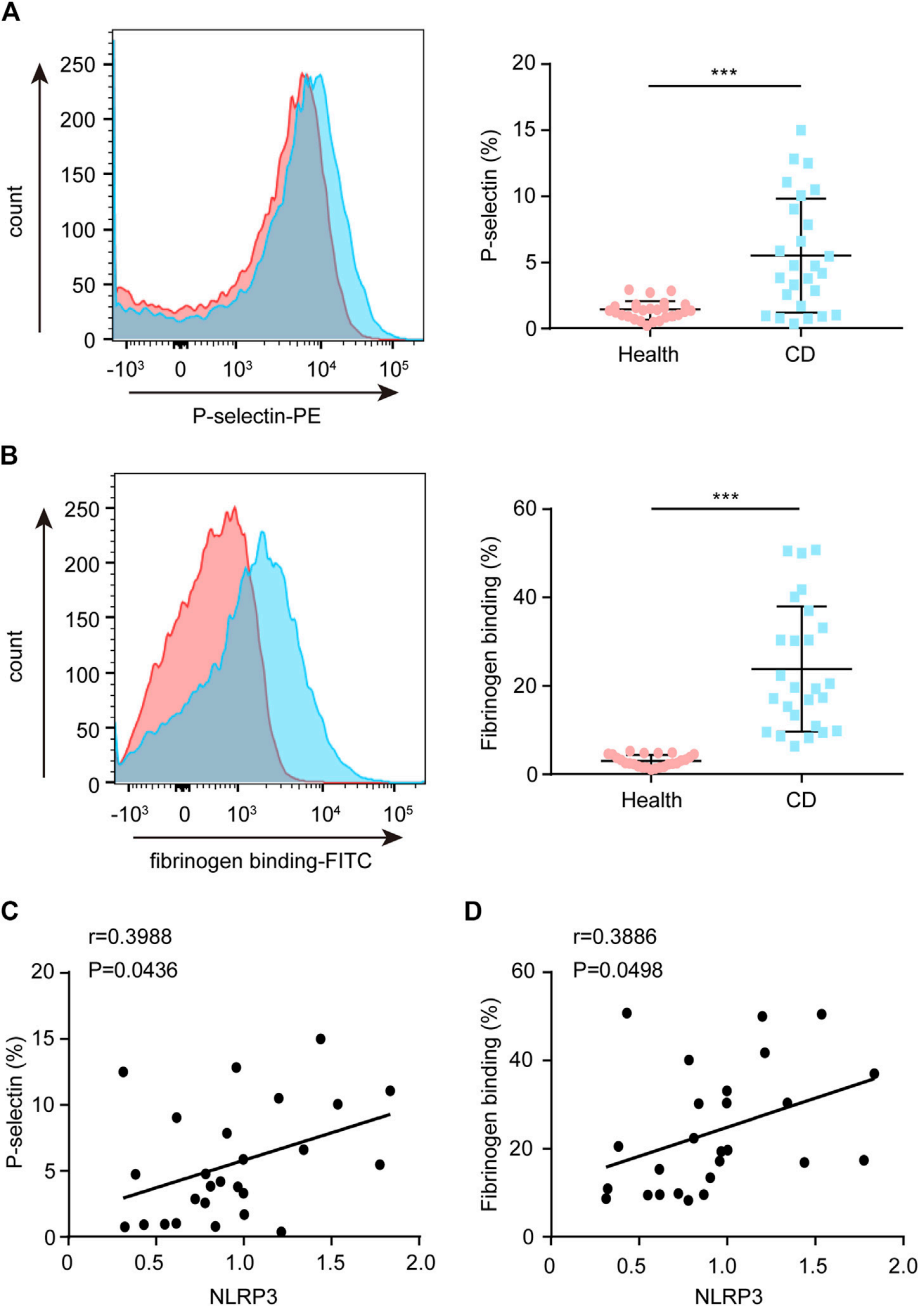
Correlation between the platelet NLRP3 protein levels and P-selectin exposure or fibrinogen binding in patients with active Crohn’s disease (CD). **(A)** P-selectin exposure was assessed by FCM in platelets from the healthy group (*n* = 26) and patients with active CD (*n* = 26). The relative quantification was at right. **(B)** Fibrinogen binding was measured by FCM in platelets from the healthy group (*n* = 26) and patients with active CD (*n* = 26). The relative quantification was at right. **(C)** The NLRP3 protein levels were positively correlated with P-selectin exposure in platelets from patients with active CD (*n* = 26). **(D)** The NLRP3 protein levels were positively correlated with fibrinogen binding in platelets from patients with active CD (*n* = 26). Health, the healthy group; CD, the patients with active CD. Data are presented as mean ± SD, ***p < 0.001.

Upon platelet activation, P-selectin will translocate to the platelet membrane. Therefore, P-selectin is a specific surface molecular marker that reflects platelet activity. Compared with the healthy group, increased P-selectin exposure was observed in platelets from patients with active CD (Figure 5A). Moreover, the NLRP3 protein levels were positively correlated with P-selectin exposure in platelets from patients with active CD (Figure 5C). In addition, upon platelet activation, inside-out signaling activates the receptor-integrin αIIbβ3, greatly enhancing their affinity for fibrinogen binding. Therefore, we investigated the ratio of fibrinogen binding. Similarly, we found that the ratio of fibrinogen binding was higher in patients with active CD than healthy subjects (Figure 5B). Moreover, the NLRP3 protein levels were positively correlated with the ratio of fibrinogen binding in platelets from patients with active CD (Figure 5D). In addition, there were significantly positive correlations between the ASC protein levels (Supplementary Figure S2A,B), the ratio of IL-1β-positive platelets (Supplementary Figure S2E,F) or ROS levels (Supplementary Figure S2G–H) and P-selectin exposure or fibrinogen binding in patients with active CD. Active caspase-1 protein levels were positively correlated with P-selectin exposure in platelets from patients with active CD (*r* = 0.7496, *p* < 0.001, Supplementary Figure S2C), but they were not associated with the ratio of fibrinogen binding (*r* = 0.1134, *p* = 0.5813, Supplementary Figure S2D). Together, these results demonstrate an association between platelet hyperactivity and ROS levels or platelet NLRP3 inflammasome components in patients with active CD.

## Discussion

**FIGURE 6:**
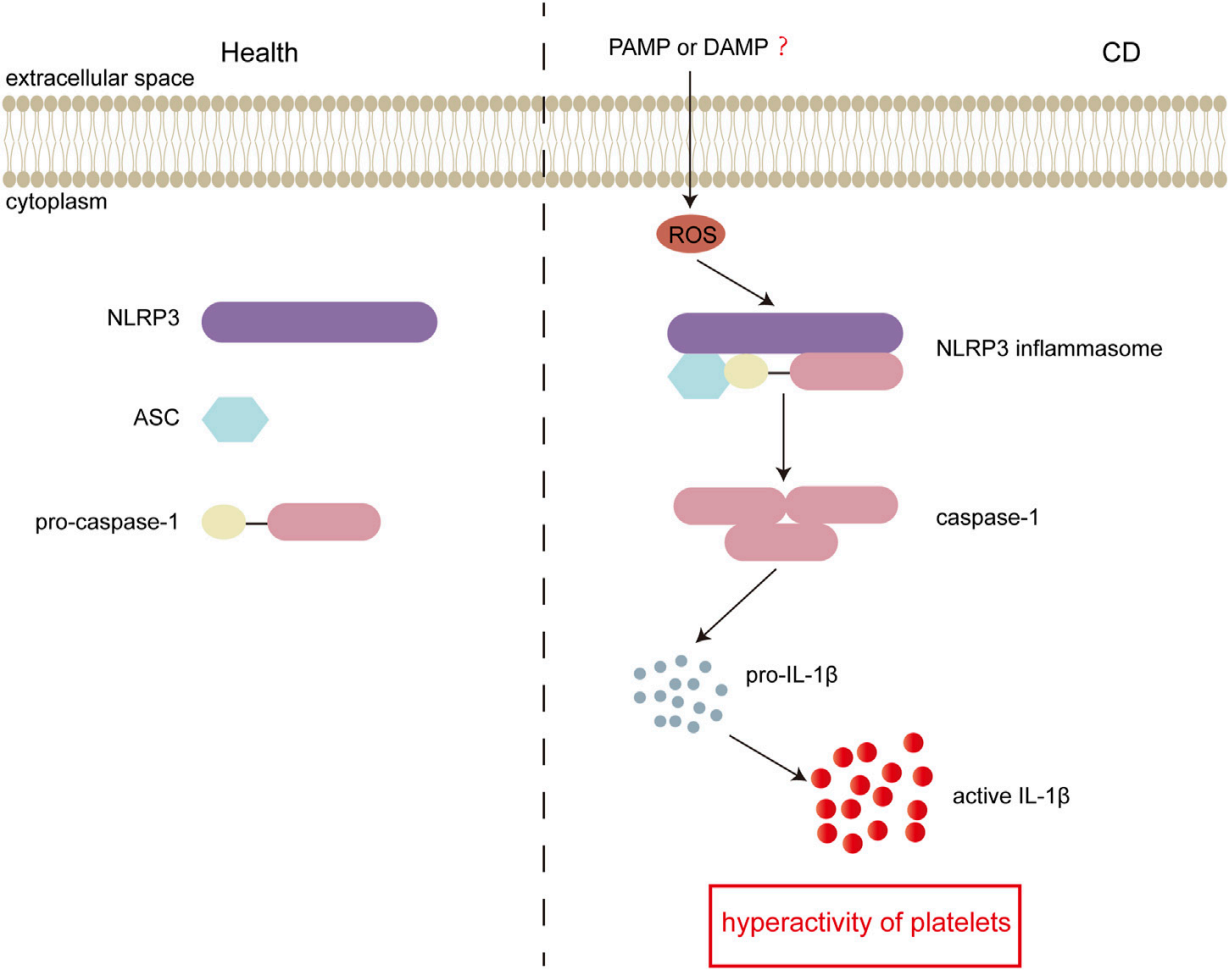
Schematic representation of inflammasome activation in active Crohn’s disease (CD). In active CD, the bacteria derived DAMPs and pathogen-associated molecular patterns (PAMPs) could enter platelets and induce generation of intracellular ROS. Elevated ROS consequently stimulates the assembly of NLRP3 inflammasome. Followed by activation of NLRP3 inflammasome, pro-caspase-1 is cleaved into active caspase-1, and consequently induces generation of interleukin-1β, which results in platelet hyperactivity. In the healthy group, there is no formation of the NLRP3 inflammasome.

CD is closely associated with platelet hyperactivity. Activated platelets participate in the pathogenesis of CD by regulating hemostasis, thrombosis, inflammation and immune signaling. However, the underlying mechanism of platelet hyperactivity in CD is not well understood. In this study, we investigated the role of NLRP3 inflammasome in platelet hyperactivity in CD. We found that the levels of the inflammasome stimulator ROS, adaptor-ASC, and sensor-NLRP3 were upregulated. In addition, platelet NLRP3 activation was enhanced in CD, and subsequently, caspase-1 was upregulated to increase interleukin-1β. More importantly, we demonstrated that the NLRP3 protein levels were positively associated with platelet activation in patients with active CD. Our results revealed that the ROS-NLRP3 inflammasome-interleukin-1β axis may contribute to platelet hyperactivity in CD (summarized in Figure 6).

Accumulating evidences have shown a close correlation between the development of CD and the assembly of NLRP3 inflammasome. Villani et al. (2009) revealed that mutations on *NLRP3* were associated with susceptibility to CD. In addition, Mao et al. (2018) found that a mutation in CARD8, a regulator of the inflammasome, could cause CD. Current studies have yielded controversial conclusions on the function of NLRP3 inflammasome in CD. Some studies suggested that a lack of caspase-1 or NLRP3 decreased the susceptibility to chemical-induced colitis, while others believed that they increased susceptibility (Allen et al., 2010; Bauer et al., 2010; Zaki et al., 2010). It is generally thought that the NLRP3 inflammasome is of great importance in the pathogenesis of CD, but its specific roles remain unclear. Here, we demonstrated increased levels of intracellular ROS and the subsequent formation of NLRP3 inflammasome in platelets from patients with active CD. Previous studies found that plasma levels of TGF-β or PDGF were significantly increased in patients with active CD, and could induce an increase in ROS in circulating cells (Takeda et al., 2014; Shi et al., 2016). We proposed that elevated plasma TGF-β or PDGF might increase ROS levels in platelets from patients with active CD, leading to platelet hyperactivity. Supporting our findings, ROS was reported to be a critical mechanism triggering the assembly of NLRP3 inflammasome in response to the damage-associated molecular patterns (DAMPs) from damaged cells (Ratajczak et al., 2019).

Recently, it has been shown that NLRP3 was expressed in platelets (Murthy et al., 2017). In *NLRP3* ^−/−^ mice, the deficiency of NLRP3 significantly impaired platelet aggregation, clot retraction, hemostasis and thrombosis. However, loss of NLRP3 did not affect platelet degranulation and outside-in signaling. In *NLRP3* ^−/−^ mice, the levels of interleukin-1β were reduced in platelets, indicating that NLRP3 was significantly conducive to the generation of interleukin-1β (Qiao et al., 2018). Interleukin-1β acts as a major mediator of innate immune responses and autoimmune inflammation, causing tissue damage in CD (He et al., 2016; Malik and Kanneganti, 2018; Chan and Schroder, 2020). Bauer et al. (2010) found that decreased interleukin-1β levels were accompanied by reduced TNBS-induced colitis and DSS-induced colitis in a mouse model. Interleukin-1β potentiated platelet secretion, aggregation, spreading and clot retraction through an autocrine loop (Qiao et al., 2018). Here, we discovered that during inflammasome assembly in platelets, caspase-1 underwent autocatalytic activation and triggered generation of interleukin-1β in patients with active CD. Recent study showed that SLE-activated platelets induced endothelial cell activation via interleukin-1β pathway (Nhek et al., 2017). Therefore, we proposed that the increased interleukin-1β from platelets of patients with active CD might also lead to the endothelial damage, which could promote the development of CD to some extent. Chanchal et al., 2020) found that IL-1β and IL-18 induced inflammatory responses, subsequently resulted in platelet activation and aggregation, thus cumulatively leading to thrombus formation. Collectively, these results indicate the NLRP3 inflammasome-interleukin-1β axis exists in platelets and may play an important role in the etiology of CD. Liu et al. (Liu et al., 2019) found that inhibition of platelet activating factor receptor (PAFR) reduced inflammation in mouse lungs after DSS challenge, and PAFR might represent a previously unappreciated mediator of secondary lung inflammation in IBD. In patients with active CD, PAFR may act as a mediator of cytokines released after platelet activation to promote disease progression. This further suggests that platelet activation plays an important role in the pathogenesis of IBD.

The incidence of thrombosis in IBD ranged from 1.2 to 7.1%, but mucosal biopsy confirmed that it was as high as 39%, and the incidence of thromboembolism was increased with an increase in inflammatory activity (Andersen and Jess, 2014). Rectal mucosal biopsy of patients with active CD showed platelet aggregation in capillaries (Orfei et al., 2019). Andoh et al. (2006) enrolled 22 healthy volunteers and 25 patients with CD treated with three platelet agonists (epinephrine, collagen and ADP), and found that almost all patients showed increased sensitivity to agonists compared to healthy participants to some extent. These results strongly suggest that platelet hyperactivity may be one of the characteristics that contributes to the increased risk of thrombosis in patients with active CD. Moreover, the platelet activation marker P-selectin has guiding significance for the judgment of the clinical condition and prognosis of CD (Fägerstam and Whiss, 2006). In this study, we found that the ratio of fibrinogen binding and P-selectin exposure was evidently upregulated in platelets from patients with active CD, which was in accordance with previous researches (Fägerstam and Whiss, 2006; Vogel et al., 2019). Notably, we observed positive correlations between ROS levels or platelet NLRP3 inflammasome components and P-selectin exposure or fibrinogen binding. A previous study found that platelet aggregation responses were enhanced in IBD patients. In their study, many cases of mild disease activity were sensitive to low concentrations of only two agonists (epinephrine/collagen or epinephrine/ADP), but all severe cases were sensitive to low concentrations of all three agonists (epinephrine/ADP/collagen) (Andoh et al., 2006). Another study found that P-selectin was a marker of severity of mucosal inflammation in ulcerative colitis (Danese et al., 2004). Therefore, there may be a positive correlation between platelet hyperactivity and the stage of CD. The higher degree of platelet activation, the more serious stage of CD.

However, this study also has some limitations. First, our study has a smaller number of cases based on 40 healthy subjects and 40 patients with active CD. In addition, we should consider the platelets in the blood vessels of the disease sites. If we can collect a certain number of precious specimens, we could study the actual behavior of platelets from a more physiological perspective, which needs further investigations.

In conclusion, we demonstrated that activation of platelet NLRP3 inflammasome might trigger enhanced interleukin-1β secretion and contribute to platelet hyperactivity in patients with active CD.

## Data Availability

The original contributions presented in the study are included in the article/Supplementary Material, further inquiries can be directed to the corresponding author.

## Supplementary Material

Supplementary Figure 1 The mRNA levels of caspase-1 in platelets from the healthy group and patients with active Crohn’s disease (CD). **(A)** The mRNA levels of caspase-1 were determined by RT-PCR in platelets from the healthy group (*n* = 17) and patients with active CD (*n* = 17). Health, the healthy group; CD, the patients with active CD. Data are presented as mean ± SD, n.s. *p* > 0.05.

Supplementary Figure 2 Correlation between ROS levels or NLRP3 inflammasome components and P-selectin exposure or fibrinogen binding in platelets from patients with active Crohn’s disease (CD). **(A)** The ASC protein levels were positively correlated with P-selectin exposure in platelets from patients with active CD (*n* = 26). **(B)** The ASC protein levels were positively correlated with fibrinogen binding in platelets from patients with active CD (*n* = 26). **(C)** The active caspase-1 protein levels were positively correlated with P-selectin exposure in platelets from patients with active CD (*n* = 26). **(D)** The active caspase-1 protein levels were not correlated with fibrinogen binding in platelets from patients with active CD (*n* = 26). **(E)** The ratio of interleukin-1β-positive platelets was positively correlated with P-selectin exposure in platelets from patients with active CD (*n* = 26). **(F)** The ratio of interleukin-1β-positive platelets was positively correlated with fibrinogen binding in platelets from patients with active CD (*n* = 26). **(G)** The ROS levels were positively correlated with P-selectin exposure in platelets from patients with active CD (*n* = 26). **(H)** The ROS levels were positively correlated with fibrinogen binding in platelets from patients with active CD (*n* = 26).
