# Supplementary figures and images for: Activation of Platelet NLRP3 Inflammasome in Crohn’s Disease
Source: Front Pharmacol. 2021 Jun 28;12:705325. doi: 10.3389/fphar.2021.705325 (PMC8273542; doi:10.3389/fphar.2021.705325)

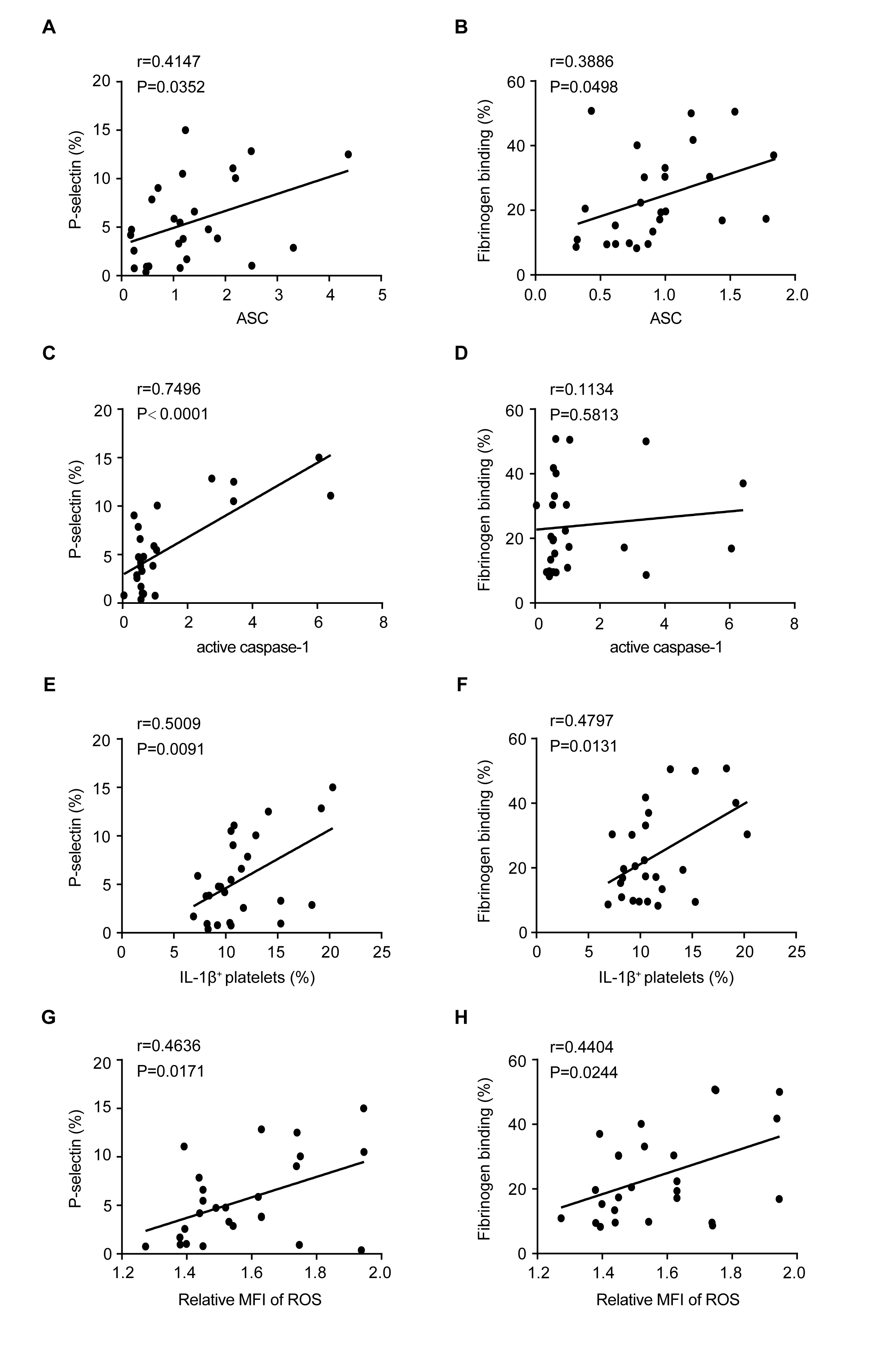

Supplement: Supplementary file 1 [file Image2.TIF]

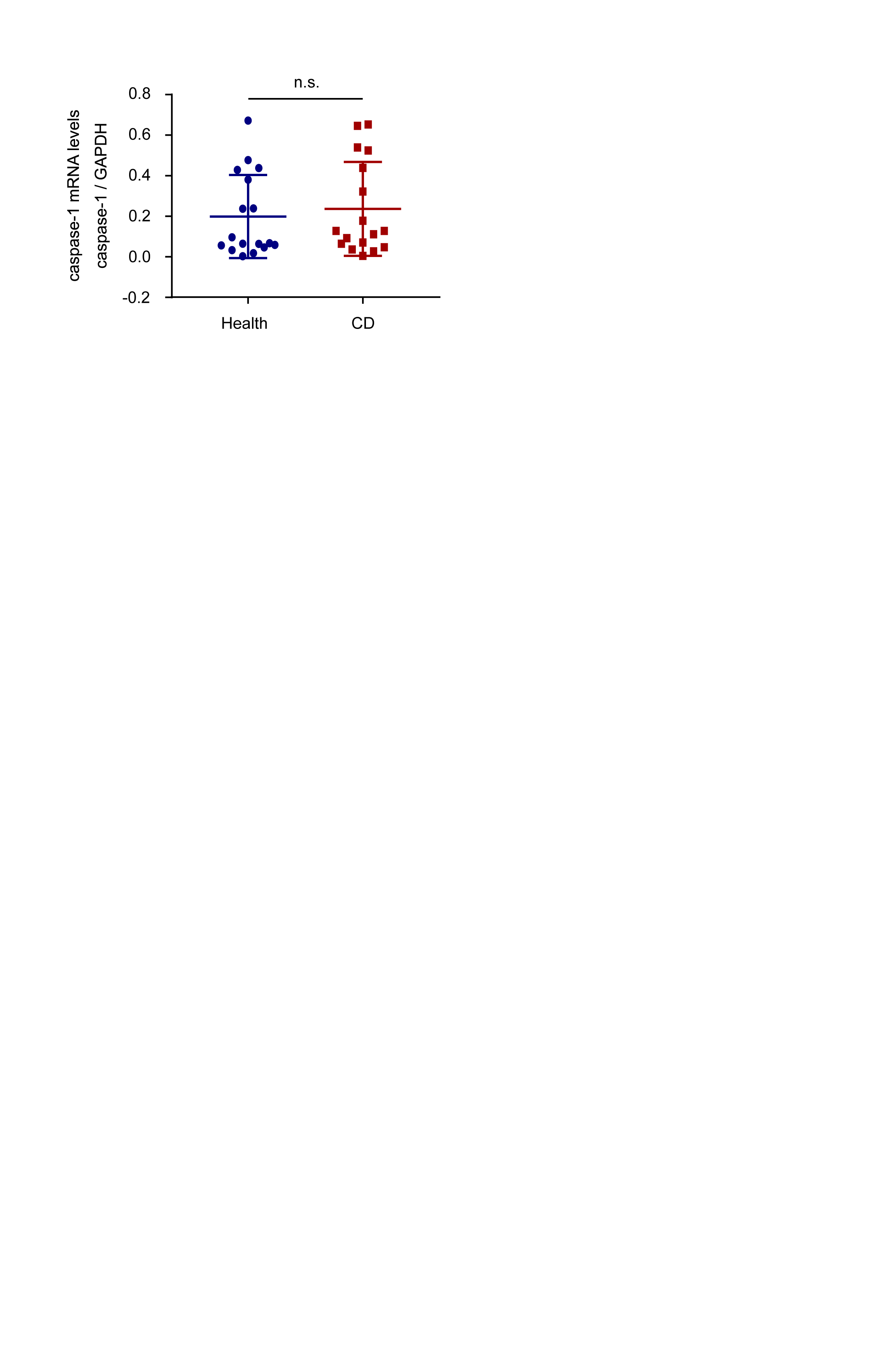

Supplement: Supplementary file 2 [file Image1.tif]
